# Combining Acute Kidney Injury with Gastrointestinal Pathology: A Clue to Acute Oxalate Nephropathy

**DOI:** 10.1155/2018/8641893

**Published:** 2018-12-23

**Authors:** Benjamin Fox, Nishkarsh Saxena, Leah Schuppener, Laura Maursetter

**Affiliations:** ^1^University of Wisconsin School of Medicine and Public Health, Madison Wisconsin, USA; ^2^Southwest Kidney Specialists, University of Wisconsin School of Medicine and Public Health, Madison, WI, USA; ^3^Division of Pathology and Laboratory Medicine, University of Wisconsin School of Medicine and Public Health, Madison, WI, USA; ^4^Division of Nephrology, University of Wisconsin School of Medicine and Public Health, Madison, WI, USA

## Abstract

Acute oxalate nephropathy (AON) is an increasingly recognized cause of acute kidney injury (AKI). Herein, we present two cases of biopsy-proven acute oxalate nephropathy in patients with gastrointestinal malabsorption, coincidentally both stemming from cholangiocarcinoma. The first is a 73-year-old male who presented with syncope and was found to have severe, oliguric AKI in the setting of newly diagnosed, nonresectable cholangiocarcinoma. The second is a 64-year-old man with remote resection of cholangiocarcinoma who presented after routine laboratory monitoring showed significant AKI. Temporary dialysis was required in both cases before renal recovery occurred. Together, these cases should increase physicians' suspicion of AON in the presence of malabsorption. By doing so, the workup of oxalate nephropathy can be expedited with prompt initiation of treatment.

## 1. Introduction

Acute oxalate nephropathy, defined as acute kidney injury (AKI) due to biopsy-proven calcium oxalate deposition, is a recognized etiology of kidney injury. Numerous studies have identified precipitants of oxalate nephropathy (AON), including ingestion of oxalate-rich foods or bariatric surgery in attempting weight loss. Prognosis is poor for patients with AON. Cohen-Bucay el al. reviewed 45 patients with AON from gastrointestinal causes and found 66% required dialysis at presentation and 42% progressed to end stage renal failure (ESRD) [1]. Fat malabsorption arising from gastrointestinal pathology is one particular common and significant risk factor of oxalate nephropathy. In this study, two cases of AON with fat malabsorption as the predominant mechanism leading to acute oxalate nephropathy will be reviewed. The first involves a patient with newly diagnosed cholangiocarcinoma yielding AON while the second concerns a patient who developed AON after receiving curative Roux-en-Y hepaticojejunostomy for cholangiocarcinoma. Our report highlights this association and serves as a valuable reminder to maintain a high degree of suspicion for AON when patients demonstrate AKI with gastrointestinal pathology.

## 2. Case 1 Presentation

A 73-year-old male veteran presented with recurrent syncope and falls. He had decreased appetite, thirst, and urine output in the setting of progressive abdominal distention, pruritus, and painless jaundice in the prior month. His past medical history was significant for obesity (BMI=40.1), hypertension, and benign prostatic hyperplasia (BPH) with pertinent medications of ibuprofen (200 mg QID), furosemide (40 mg BID), losartan, doxazosin, and finasteride. A detailed dietary history prior to admission was lacking. Physical exam was notable for diffuse jaundice, abdominal distention, and severe mid-thoracic back pain later confirmed to be T6/T7 vertebral fractures.

Initial laboratory results showed stage 3 AKI with serum creatinine (SCr) elevated to 8.98 mg/dL from a baseline of 1.04 mg/dL. Supporting laboratory findings included elevations in phosphorus (7.6 mg/dL), parathyroid hormone (319.7 pg/mL), and low calcium (7.6 mg/dL), ionized calcium (0.93 mmol/L), albumin (2.8 g/dL), and 25-hydroxy vitamin D (16.4 ng/mL). There was an anion gap (21 mEq/L) with pH= 7.145 confirmed with arterial blood gas. Jaundice workup revealed an obstructive pattern with elevated total bilirubin (5.4 mg/dL), direct bilirubin (3.7 mg/dL), AST (79 U/L), ALT (140 U/L), alkaline phosphatase (392 U/L), GGT (214 U/L), and lipase (690 U/L). Soon after admission, he developed hypotension necessitating vasopressors and broad-spectrum antibiotics for presumed septic shock. AKI workup revealed deteriorating kidney function and development of anuria, requiring intermittent hemodialysis.

**Figure 1:**
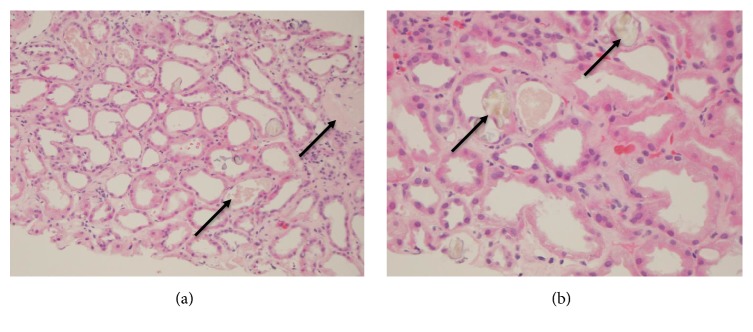
(a) Renal biopsy light microscopy (stain, hematoxylin and eosin) showing renal tubules with severe acute injury, epithelial flattening and diffuse deposition of oxalate crystals (arrow) within the cortex and medulla. (b) Renal tubules containing numerous oxalate crystals (arrow).

The etiology of renal failure remained unclear. Urine testing showed nephrotic range proteinuria, many bacteria, no red blood cells, no crystals, few hyaline casts, and no granular casts. A renal biopsy was performed and showed diffuse calcium oxalate crystal deposition with severe acute tubular injury (ATN) and mild interstitial fibrosis (Figure 1(a)). An evident cause of oxalate nephropathy was unclear, although it was suspected that pancreatic insufficiency leading to fat malabsorption and increased intestinal absorption of oxalate were responsible. Fecal elastase returned low at 54 *μ*g Elastase/g stool to support pancreatic insufficiency while abdominal computed tomography (CT) without contrast showed exocrine atrophy of the pancreas. Serum oxalate was 3.6 mmol/L (normal range 1.0-3.0 mmol/L). A low-fat, low-oxalate diet with calcium citrate and pancrelipase supplementation was started.

Concurrently, a jaundice workup began with abdominal ultrasound which showed hepatomegaly, splenomegaly, numerous gallstones, and no inferior vena cava or portal circulation thrombosis. Of note, the common bile duct was not visibly dilated, raising concern for an upstream malignant stricture. MRCP showed marked dilation of the left intrahepatic biliary tree with abrupt termination and mild dilation of the right intrahepatic biliary tree, further raising suspicion of malignancy. ERCP with stenting to the right intrahepatic biliary tree duct was performed. Cytology, a markedly elevated CA 19-9, and imaging confirmed the diagnosis of cholangiocarcinoma (Klatskin tumor). Medical oncology discussed chemotherapy and radiation as alternative treatments, but the patient elected to enter hospice. His kidney function ultimately improved with return of nonoliguric urine output, cessation of dialysis, and improved creatinine to 2.45mg/dL and eGFR of 26 ml/min/1.73m^3^ at discharge.

## 3. Case 2 Presentation

A 64-year-old male presented from clinic after routine lab monitoring showed new AKI and hyperkalemia. He was asymptomatic other than fatigue with no prior history of kidney disease. Past medical history was significant for hypertension, gout, and cholangiocarcinoma diagnosed in 2003. His cancer was in remission after chemotherapy and surgical interventions including complete excision of the extrahepatic biliary tree, Roux-en-Y hepaticojejunostomy, and cholecystectomy. He developed chronic pancreatitis and insulin-dependent diabetes postoperatively. Pertinent medications included losartan, triamterene-hydrochlorothiazide, and insulin. Admission vitals were notable for BP 165/71 and he was euvolemic on physical exam.

Laboratory workup revealed stage 3 nonoliguric AKI with serum creatinine of 4.61 mg/dL elevated from a stable baseline of 0.94 mg/dL. Supporting labs included elevations in potassium (5.4 mEq/dL), phosphorus (6.9 mg/dL), uric acid (10.5 mg/dL) and low bicarbonate (16 mEq/L), normocytic anemia (hemoglobin 9.9 g/dL), and hypoglycemia (blood sugar 32 mg/dL). CA19-9 was elevated to 51, but this was stably elevated and not felt secondary to signify recurrent disease. His hemoglobin A_1_C was 5.4%. Urine analysis showed 2-3 WBC/hpf. The etiology of his AKI was unclear but AIN was considered given his use of triamterene and leukocytes on urine microscopy.

**Figure 2:**
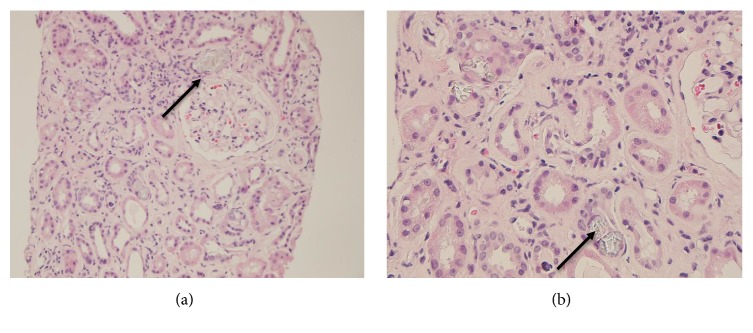
(a) Renal biopsy light microscopy (hematoxylin and eosin) showing renal tubules with severe acute injury, epithelial flattening and diffuse deposition of oxalate crystals within the cortex and medulla. (b) Renal tubules containing numerous oxalate crystals.

Subsequent renal biopsy showed severe, chronic active interstitial nephritis, severe interstitial fibrosis and tubular atrophy, and oxalate nephropathy (Figure 2). The oxalate nephropathy was believed secondary to enteric hyperoxaluria due to fat malabsorption from chronic pancreatitis and Roux-en-Y bypass. Followup 24-hour urine collection showed high oxalate excretion (90 mg) with low calcium (53 mg) and citrate (<28 mg) consistent with hyperoxaluria. 24-hour fecal fat was elevated at 26.3 g suggesting pancreatic insufficiency. Intermittent hemodialysis was initiated. He was started on a low oxalate diet with supplementation of pancrelipase, calcium citrate, and potassium citrate. Serum oxalate improved from 11.0 *μ*mol/L to 7.9 *μ*mol/L with these interventions. Serum creatinine peaked to 5.37 mg/dL before plateauing with cessation of dialysis. After further education and diet changes, his creatinine improved and plateaued to 3.57 mg/dL two years after admission.

## 4. Discussion

Oxalate is a small divalent anion and end product of metabolism of multiple precursors including amino acids, glyoxylate, and ascorbic acid (Vitamin C) [2]. Endogenous hepatic metabolism of these precursors contributes to 90% of the total body load of oxalate while the remaining 10% is from enteric absorption of free oxalate [3]. Oxalate is eliminated from the body solely by renal excretion as it is freely filtered by glomeruli [4]. When excess renal excretion of oxalate occurs in conditions of supersaturation, oxalate readily binds with divalent cations such as calcium and crystalizes. Calcium oxalate precipitation then yields a spectrum of renal disease: from nephrolithiasis to acute oxalate nephropathy (AON), gradual renal interstitial deposition termed renal oxalosis, and even systemic oxalosis or the body-wide deposition of calcium oxalate [2].

Risk factors for oxalate nephropathy of any kind can be divided into three categories: (1) primary hyperoxaluria (PH) which are rare inherited enzyme defects upregulating hepatic production of oxalate, (2) after kidney transplantation when the burden of oxalate which has accumulated during CKD/ESRD needs to be excreted to achieve equilibrium, and (3) increased gastrointestinal absorption of oxalate. Enteric hyperoxaluria is further subdivided into excess ingestion of large loads of oxalate-rich foods and/or increased small bowel wall permeability to oxalate uptake. Enteric hyperoxaluria leading to AON has numerous well-documented causes, including consumption and juicing of oxalate-rich foods (spinach, parsley, peanuts, soy, and star fruit) in weight loss attempts, high intake of vitamin C supplementation, low vitamin B_6_ intake, toxic ingestions of ethylene glycol (antifreeze), and multiple causes of fat malabsorption (chronic pancreatitis, gastric bypass, medications inhibiting pancreatic lipase, and various causes of colitis including inflammatory bowel disease) [2, 5–9]. Clostridium colitis infection has also been implicated in AON as antibiotic therapy is known to reduce the intestinal bacteria Oxalobacter formigenes capable of metabolizing oxalate, thereby increasing available oxalate for absorption [1]. A final interesting mechanism may involve the role of prostaglandins in decreasing calcium oxalate adhesion to renal epithelial cells [2, 10, 11]. As NSAIDs downregulate prostaglandin production, it is feasible NSAIDs predispose to greater likelihood of stone formation. This mechanism may have contributed to AON in our first patient given his frequent NSAID use.

It is difficult to precisely determine one distinct cause of oxalate nephropathy as there are often multiple contributing factors in renal oxalosis [5, 12]. Getting et al. reviewed 65 biopsy-proven cases of AON and found that 29 (45%) had at least two possible reasons for oxalate nephropathy [5]. In a majority of cases, existing CKD and prior renal transplant were established risk factors to which another precipitant was added.

Our first patient's AON was likely multifactorial with gastrointestinal causes predominating. Fat malabsorption from pancreatic insufficiency is likely the first contributing mechanism of increased intestinal absorption of oxalate. Our patient's low fecal elastase (54 *μ*g Elastase/g stool) and CT finding of exocrine pancreatic atrophy make pancreatic insufficiency likely albeit he did not endorse classic symptoms of steatorrhea and weight loss. Pancreatic insufficiency leads to an increased intestinal concentration of undigested fatty acids, which binds preferentially to calcium rather than oxalate. This, in turn, increases free oxalate absorption as less calcium is available to bind oxalate, a complex that is not absorbed. The second related mechanism is reduced bile salt delivery to the intestine from cholangiocarcinoma yielding biliary tree obstruction. With decreased bile salts, intestinal fatty acids undergo inadequate emulsification to further promote their complex formation with dietary calcium rather than oxalate [13]. Emmett et al. showed decreased oxalate uptake when supplementing human subjects with conjugated bilirubin, suggesting this is a biologically plausible mechanism [14]. Taken together, without sufficient pancreatic lipase and bile acids to digest and emulsify fats, respectively, fatty acids bind preferentially with calcium rather than oxalate to risk enteric hyperoxaluria [2].

Fat malabsorption was also a contributor for AON in the second patient. Significant dietary changes due changes in his living situation exposed him to a variety of different foods, some of which were high in oxalate. This patient highlights the need for continued education of a low-oxalate diet given his kidney function worsened after discharge due to both lack of understanding of the importance of his medications and lack of low-oxalate foods. Since becoming educated, he has remained off dialysis.

In conclusion, these cases illustrate that AKI in patients with gastrointestinal disease should raise suspicion for AON. Specifically, nephrologists should suspect AON when other causes are less likely or there is lack of evidence of other diagnoses. This can prompt more liberal biopsy patterns which were necessary in both of these cases to make the diagnosis. In addition, gastroenterologists need to have a working suspicion of AON as they will be the first line of defense for recognizing AKI in susceptible patients and educating patients with the risk of malabsorption that AON can occur. AON requires swift adjustments in treatment to keep patients away from needing chronic dialysis; therefore nephrologists, gastroenterologists, and patients need to be aware.
